# Phenotypic and genotypic characterization of commensal staphylococci isolated from young volunteers in Alexandria, Egypt

**DOI:** 10.1038/s41598-024-60924-8

**Published:** 2024-06-27

**Authors:** Aisha Hamdy, Tessa Marciniak, Mustafa Alseqely, Wilma Ziebuhr, Elsayed Abouelmagd, Alaa Abouelfetouh

**Affiliations:** 1 Department of Microbiology and Immunology, Faculty of Pharmacy, Alexandria University, Alexandria, Egypt; 2 Institute for Molecular Infection Biology, University of Würzburg, Würzburg, Germany; 3 Department of Microbiology and Immunology, Faculty of Pharmacy, Arab Academy for Science, Technology, and Maritime Transport, Alamein Branch, Alexandria, Egypt; 4 Department of Microbiology and Immunology, Faculty of Pharmacy, Alamein International University, New Alamein, Egypt

**Keywords:** *S. aureus*, SOSA, Nasal colonization, Antibiotic resistance, Egypt, Community acquired infections, Bacterial infection, Antibiotics, Clinical microbiology

## Abstract

Nasally colonized staphylococci carry antibiotic resistance genes and may lead to serious opportunistic infections. We are investigating nasal carriage of *Staphylococcus aureus* and Staphylococci other than *S. aureus* (SOSA) among young volunteers in Egypt to determine their risk potential. Nasal swabs collected over 1 week in June 2019 from 196 volunteers were cultured for staphylococcus isolation. The participants were interviewed to assess sex, age, general health, hospitalization and personal hygiene habits. Identification was carried out using biochemical tests and VITEK 2 automated system. Disc diffusion and minimum inhibitory concentration tests were performed to determine antibiotic susceptibility. Screening for macrolide resistance genes (*ermA, ermB, ermC, ermT* and *msrA*) was performed using polymerase chain reaction. Thirty four *S. aureus* and 69 SOSA were obtained. Multi-drug resistance (MDR) was detected among most staphylococcal species, ranging from 30.77% among *S. hominis* to 50% among *S. epidermidis*. Phenotypic resistance to all tested antibiotics, except for linezolid, was observed. Susceptibility to rifampicin, vancomycin and teicoplanin was highest. *ermB* showed the highest prevalence among all species (79.41% and 94.2% among *S. aureus* and SOSA, respectively), and constitutive macrolide-lincosamide-streptogramin B (MLS_B_) resistance was equally observed in *S. aureus* and SOSA (11.11% and 16.22%, respectively), whereas inducible MLS_B_ resistance was more often found in *S. aureus* (77.78% and 43.24%, respectively). The species or resistance level of the carried isolates were not significantly associated with previous hospitalization or underlying diseases. Although over all colonization and carriage of resistance genes are within normal ranges, the increased carriage of MDR *S. aureus* is alarming. Also, the fact that many macrolide resitance genes were detected should be a warning sign, particularly in case of MLS_B_ inducible phenotype. More in depth analysis using whole genome sequencing would give a better insight into the MDR staphylococci in the community in Egypt.

## Introduction

Staphylococci are common infectious agents, with *Staphylococcus aureus* being the prominent species whose pathogenic potential can range from mild superficial skin infections to severe pneumonia and life-threatening systemic diseases such as septicemia^1^. Staphylococcal species other than *S. aureus* (SOSA), formerly grouped as coagulase-negative staphylococci, play a role as human pathogens as well, particularly in the context of nosocomial and foreign-body-associated infections, where they pose a serious threat, especially to immunocompromised patients and premature infants^2^. A common theme in staphylococcal infections, both in the community and in health care settings, is the pronounced capacity of staphylococci to readily acquire a multitude of antibiotic resistance (ABR) determinants, often leading to the emergence of multi-drug resistant (MDR) strains. A prime example for ABR staphylococci are methicillin resistant *S. aureus* (MRSA) which evolved through acquisition of *mec* genes, mediating resistance to all beta-lactam antibiotics^3^. MRSA, which often exhibit additional ABRs to other antibiotic classes, are prevalent worldwide and represent a significant public health issue, which caused more than 100,000 deaths in 2019 alone^4^. However, apart from their importance as pathogens, staphylococci are primarily commensals that have their natural habitat on human skin and mucous membranes (such as the anterior nares), where they make up a significant part of the healthy microbiota. *Staphylococcus epidermidis*, *Staphylococcus hominis* and a number of other SOSA species are typical human skin commensals^2,5,6^. In addition, 30% of the healthy human population is permanently colonized by *S. aureus* without showing clinical symptoms^7^. Of note, the majority of both *S. aureus* and SOSA infections are caused by strains that were originally present on the patient's skin, suggesting that colonization is a prerequisite and source for (later) infection^8–10^. Also, commensal microbiota are increasingly recognized as reservoirs in which ABR genes are exchanged and in which MDR isolates can evolve and spread^11^. Therefore, studying commensal staphylococci and their ABR patterns is currently in the focus to both assess the infection risk by MDR staphylococci and to gain insight into the ABR genes currently circulating in staphylococcal populations in various habitats. Most work in the field, also on the African continent, has been done on staphylococci originating from patients and staff in health care facilities^12,13^. Thus, a most recent meta-analysis from Egypt revealed that the MRSA prevalence is high (i.e. 63%) among infection-associated clinical *S. aureus* isolates from humans^14^. However, we currently lack comparable data on staphylococci in the community. With this study, we aim to fill this knowledge gap by investigating the species composition and ABR situation in colonizing staphylococci isolated from young volunteers in the urban region of Alexandria, Egypt. We specifically focus on colonization rates by *S. aureus* and SOSA in this group of individuals as well as on resistance rates to macrolides which are among the most commonly prescribed antibiotics in Egypt^15,16^. Finally, we ask the question if personal hygiene habits have an influence on staphylococcal skin colonization and ABR rates.

## Materials and methods

### Study population

This is a cross-sectional study where nasal swabs were collected from both anterior nares of young pharmacy students, at Alexandria University over a week in June 2019. At the time of swab collection, the students filled a survey to assess their personal hygiene habits, diseases they are suffering from, recurrent infections as skin infections, and the times of hospitalization in the previous year.

The collected swabs were inoculated into nutrient broth (Himedia, India) and incubated overnight at 37 °C. The cultures were then plated onto nutrient agar (Oxoid Ltd; Basingostok; Hampshire, England) for colony isolation. Staphylococci were identified based on conventional methods, including microscopic examination, growth onto mannitol-salt agar (Oxoid Ltd; Basingostok; Hampshire, England) and deoxyribonuclease (DNase) agar (Lab M Ltd; Heywood, Lancashire, United Kingdom), catalase test using 3% hydrogen peroxide (H_2_O_2_) reagent—10 volume—(LUNA cosmetics company, Egypt) and tube coagulase test using coagulase rabbit plasma (Himedia, India). Identification to the species level was performed by VITEK 2 automated system (bioMerieux, France) for Gram-positive identification test (GPI).

### Inclusion and exclusion criteria

Pharmacy students present on campus at the time of the survey were approached, and the study was explained. All students who agreed to join the study and signed an informed consent were recruited. Refusal to join the study and/or to sign the informed consent form was the only exclusion criterion applied.

### Antimicrobial susceptibility testing

Antimicrobial susceptibility tests were performed using disk diffusion on Mueller–Hinton agar (Oxoid Ltd; Basingostok; Hampshire, England), according to the Clinical and Laboratory Standards Institute (CLSI 2020)^17^, (CLSI 2007 for vancomycin)^18^ and European Committee on Antimicrobial Susceptibility Testing (EUCAST 2023)^19^ guidelines. The following antibiotic disks (Oxoid, UK, and Himedia, India) were used: azithromycin (AZM, 15 µg), cefoxitin (CX, 30 µg), chloramphenicol (C, 30 µg), clindamycin (CD, 2 µg), co-trimoxazole (COT, 25 µg), erythromycin (E, 15 µg), fusidic acid (FC, 10 µg), gentamicin (GEN, 10 µg), linezolid (LZ, 30 µg), moxifloxacin (MO, 5 µg), mupirocin (MUP, 200 µg), rifampicin (RIF, 5 µg), teicoplanin (TEI, 30 µg), tetracycline (TE, 30 µg) and vancomycin (VA, 30 µg).

The minimum inhibitory concentrations of azithromycin (as Zithromax^®^, Pfizer), cefoxitin (as Primafoxin^®^, ZAD), chloramphenicol (as IsoptoFenicol^®^, Alcon), doxycycline (as Vibramycin^®^, Pfizer), fusidic acid (a gift from Orchidia pharmaceutical industries), gentamicin (as Epigent^®^, EIPICO), linezolid (as Voxazoldin^®^, Rofabiogen), and vancomycin (as Vancomycine^®^, Mylan S.A.S) were determined using agar dilution method following the guidelines of CLSI 2020^17^ and EUCAST 2023^19^. MIC_50_ and MIC_90_ were calculated according to Schwarz et al.^20^. Except for fusidic acid, all antibiotics were purchased on the Egyptian market. Multi-drug resistant isolates were defined as isolates showing resistance towards at least one antimicrobial agent from three or more categories^21,22^.

### D-test

In order to investigate the inducibility of clindamycin resistance, erythromycin-resistant isolates were subjected to ‘D test’. In this test, erythromycin (15 μg) and clindamycin (2 μg) discs were placed at a distance of 15 mm apart (edge to edge) on a Mueller Hinton agar plate previously inoculated with 0.5 McFarland bacterial suspension. Following overnight incubation at 37 °C, flattening of the inhibition zone (formation of D shape) around the clindamycin disc in the area between the two antibiotic discs was analysed, indicating inducible clindamycin resistance^23,24^.

### Characterization of macrolide resistance genes

For genomic DNA extraction, a few colonies were suspended in 100 µl of sterile distilled water, boiled for 30 min at 95 °C and immediately cooled at − 20 °C for 30 min prior to centrifugation (microcentrifuge, Hettich, Germany) at 14,000 rpm for 10 min. The supernatant containing genomic DNA was transferred to a new Eppendorf tube and was used for subsequent polymerase chain reaction (PCR) amplification of the target genes using PCR thermal cycler, (Perkin Elmer, USA). The sequences of the primers used to detect *ermC, ermA, ermT, ermB* and *msrA* together with annealing temperatures are listed in (Supplementary file 1). The PCR conditions were as follows: an initial denaturation at 95 °C for 1 min followed by 35 cycles of denaturation at 95 °C for 15 s, annealing for 15 s, and extension at 72 °C for 10 s, then a final extension step at 72 °C for 10 min.

### Statistical analysis

The collected data were analyzed using GraphPad Prism (version 9.5.1) (Dotmatics, Boston, Massachusetts, USA) or Statistical Package for Social Science (SPSS v25.0) (IBM, USA). Fisher's exact test was performed on significance level of 0.05.

### Ethical approval and consent to participate

The study was approved by The Research Ethics Committee at the Faculty of Pharmacy, Alexandria University prior to study commencement and the Ethics Committee of Alexandria University, Faculty of Medicine under IRB number: 00012098 and the Federal Wide Assurance FWA number: 00018699 (Date: 9/4/2023) (http://www.hhs.gov/ohrp/assurances/index.html). All potential study participants were provided with a participant information sheet prior to taking part in the study, and informed consent was obtained from all participants for the collection of swabs and use of their data before beginning the study. All experiments were conducted according to the relevant guidelines and regulations.

## Results

### Sample collection and species identification

**Figure 1:**
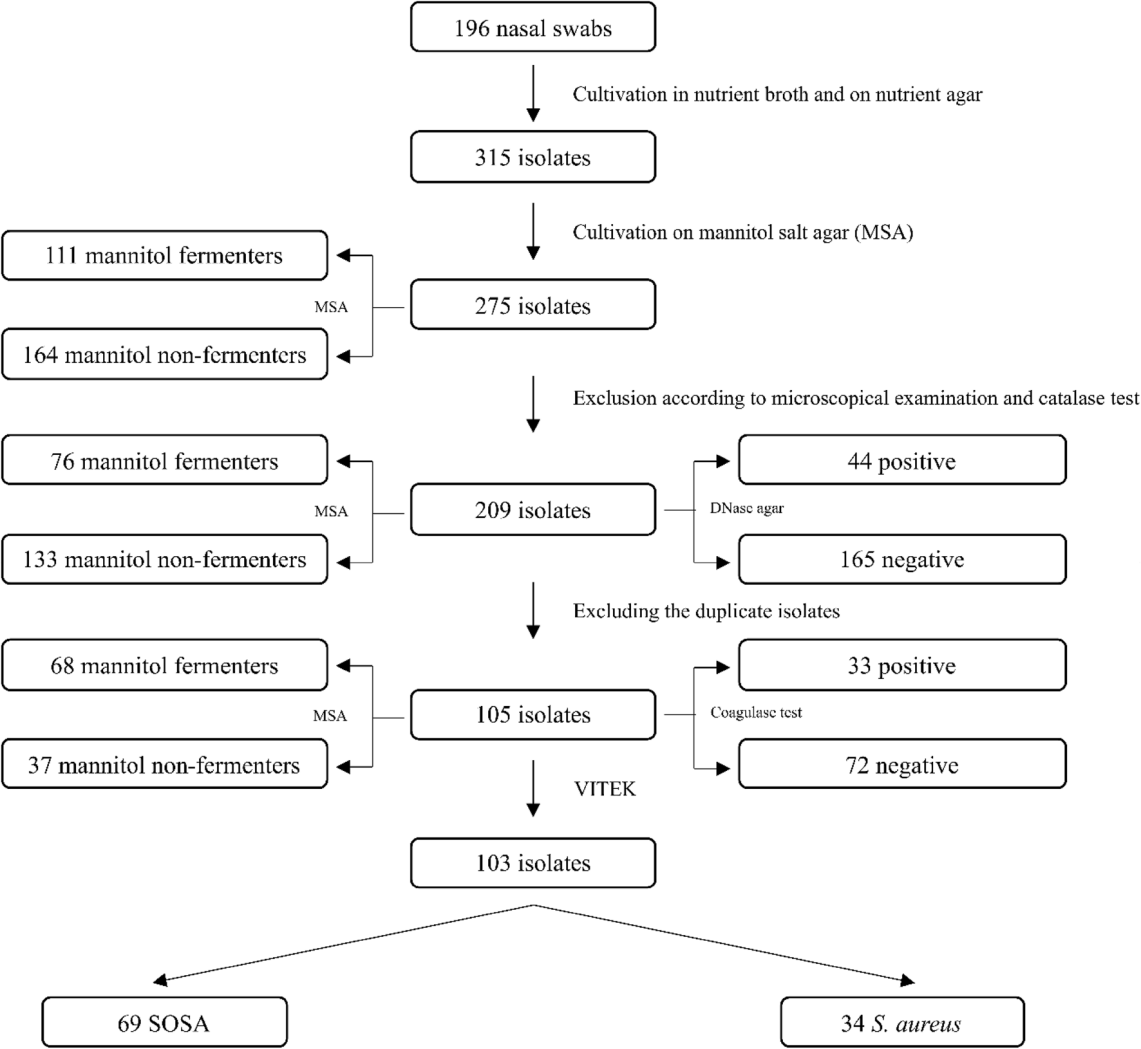
Flowchart of isolate identification process.

**Figure 2:**
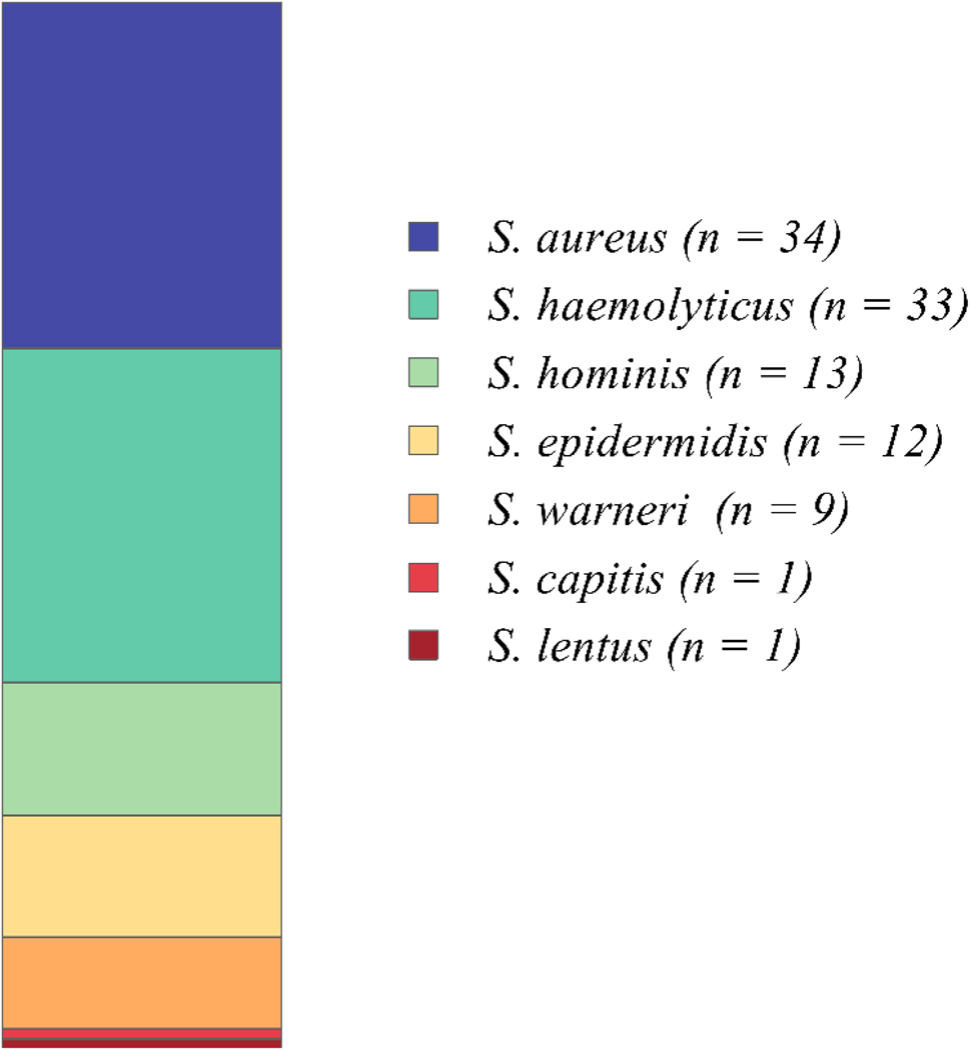
Species distribution among the isolated staphylococci.

Nasal swabs were collected from 196 young pharmacy students aged 19–23 years. Processing of the swabs according to the scheme shown in Fig. 1 yielded a total of 103 staphylococcal isolates which were obtained from 86 participants (Fig. 1, Supplementary file 2). The assignment of the isolates as staphylococci was based on microscopic examination, catalase production and growth on mannitol salt agar (Supplementary files 3 and 4). *S. aureus* isolates were identified by coagulase tests, fermentation of mannitol as well as by growth on DNase agar. Final species determination was performed using the VITEK 2 system, identifying 34 (33.01%) *S. aureus* and 69 (66.99%) Staphylococci other than *S. aureus* (SOSA) (Fig. 1, Supplementary files 3 and 4). The SOSA isolates belonged to six species: *S. haemolyticus* (*n* = 33, 32.04%), *S. hominis* subspecies *hominis* (*n* = 13, 12.62%), *S. epidermidis* (*n* = 12, 11.65%), *S. warneri* (*n* = 9, 8.74%), *S. capitis* and *S. lentus* (*n* = 1, 0.97%, each) (Fig. 2, Table 1).

**Table 1:** Participants' demographics showing relationships between species and nose washings or showering frequency.

Species	No. of isolates (%)	Average age (years)	Male:female ratio (NA)	Average number of nose washings (ablutions) daily	*p* values*	Average number of showers weekly	*P* values*
*S. aureus*	34 (33.01)	21.5	14:14 (6)	4.63	0.25	4.08	1
*S. haemolyticus*	33 (32.04)	21.9	16:14 (3)	4.46	0.26	4.2	0.37
*S. hominis*	13 (12.62)	22	4:9	3.85	0.77	5	0.23
*S. epidermidis*	12 (11.65)	21.45	5:6 (1)	4.6	0.19	4.54	0.75
*S. warneri*	9 (8.74)	21.8	4:5	4.33	0.16	5.44	0.18
*S. capitis*	1 (0.97)	NA	NA	NA	NA	NA	NA
*S. lentus*	1 (0.97)	22	0:1	1	0.42	3	1
**Total**	103	21.8	43:49 (11)	4.3	–	4.5	–

*NA* data not available. *p < 0.05 was considered as significant.

### Participant characteristics and staphylococcal colonization

Of the 196 participants originally enrolled in the study, staphylococci were recovered from 86 individuals. The participants’ female to male ratio was 42:33, with 11 participants not providing information. According to self-disclosure (Supplementary file 2), 20/86 participants (23.26%) suffered from recurrent skin infections, and 11/86 (12.79%) had been hospitalized in the previous year. 4/86 (4.65%) participants reported suffering from allergic reactions, including asthma, and 11/86 (12.79%) participants experienced recurrent infections, mostly of the respiratory tract (Supplementary file 2). Further, the subjects were asked about their personal hygiene habits in order to assess a putative correlation with colonization by *S. aureus* and SOSA species. The participants washed their nose between 1 to 7 times (an average of 4.3 times) per day and showered between 1 to 8 times (an average of 4.5 times) weekly.

A total of 30/86 subjects were colonized by *S. aureus*, with 12/30 (40%) of the isolates being MRSA, resulting in a total *S. aureus* colonization rate of 34.89% and an MRSA carriage rate of 14%. Two of the 11 individuals (18.18%) who reported a hospital stay in the previous year carried an *S. aureus* from which one isolate was an MRSA (Supplementary files 2 and 5). Hospitalization did not significantly influence *S. aureus* colonization (*p* = 1). The majority of *S. aureus* nasal carriers (24/30, 80%) were exclusively colonized by *S. aureus*, while six individuals additionally harbored SOSA. Among the 12 MRSA carriers, two were found to be co-colonized by SOSA. Exclusive colonization with SOSA was found in 56/86 (65.12%) of the participants, with the majority (50/56; 89.29%) being colonized with only one species or strain, while 6/56 (10.71%) of the subjects carried more than one SOSA species or different strains of the same species (Supplementary file 2). Of the 20/86 (23.26%) individuals with recurrent skin infections, 7/20 (35%) were colonized by *S. aureus*, with 4/7 (57.14%) isolates being MRSA (Supplementary file 5). However, there was no statistically significant correlation between skin infection and *S. aureus* colonization (*p* > 0.5; *ns*). Also, analysis of the data with respect to other reported previous illnesses did not reveal significant correlations with *S. aureus* and/or MRSA carriage. Likewise, presence of a distinct staphylococcal species was neither correlated with gender (*p* > 0.5; *ns*) nor the frequencies of nasal washings or frequencies of weekly showers (*p* > 0.5; *ns*) (Table1).

### Antimicrobial susceptibility testing and multidrug-resistance

**Figure 3:**
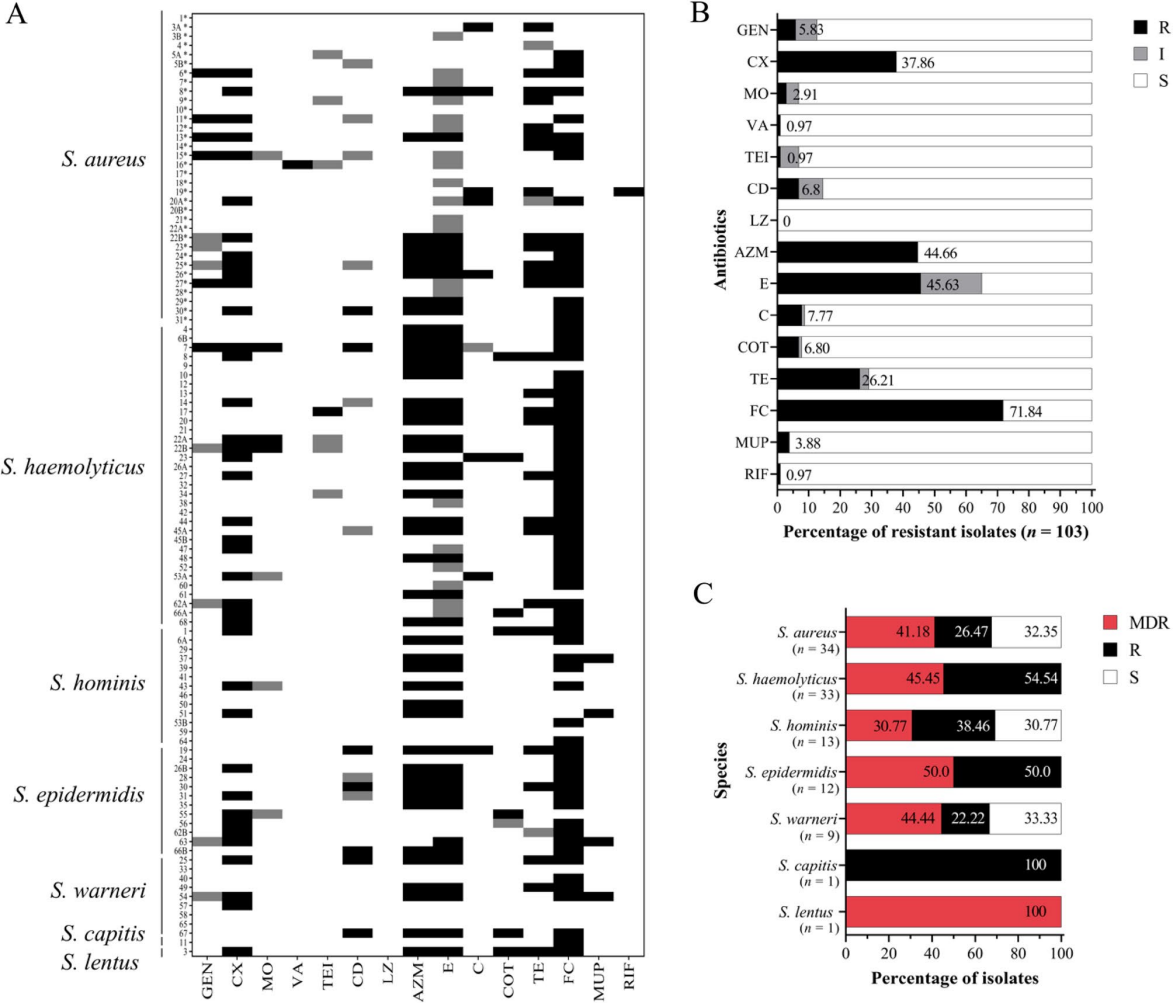
(**A**) Antibiotic resistance patterns of individual staphylococci. Heatmap represents resistance (R) in black, intermediate (I) in gray and susceptible (S) results in white, determined using disc diffusion assay. GEN—gentamicin, CX—cefoxitin, MO—moxifloxacin, VA—vancomycin, TEI—teicoplanin, CD—clindamycin, LZ—linezolid, AZM—azithromycin, E—erythromycin, C—chloramphenicol, COT—co-trimoxazole, TE—tetracycline, FC—fusidic acid, MUP—mupirocin, RIF—rifampicin. (**B**) Distribution of resistance profiles to the tested antibiotics. Numbers in graph show percentage of resistant isolates. Total number of isolates *n* = 103. (**C**) Distribution of resistance levels among individual species. Percentage of multi-drug resistant (MDR; red), resistant (R; black) and susceptible (S; white) isolates per species. Percentages given for MDR, R and S.

Resistance towards the tested antibiotics was widespread among all isolates (Fig. 3, Supplementary files 5 and 6). *S. aureus* isolates (*n* = 34) showed high resistance rates to fusidic acid (52.94%), tetracycline (38.24%) as well as to erythromycin and azithromycin (26.47%, each). Based on cefoxitin resistance screening^17^, 35.29% (12/34) of the *S. aureus* isolates were identified as MRSA. Among SOSA, *S. epidermidis* isolates (*n* = 12) displayed highest resistance levels to cefoxitin (50%), clindamycin (25%), erythromycin (66.67%) and azithromycin (58.33%) (Fig. 3). Moxifloxacin resistance was only observed among *S. haemolyticus* isolates (*n* = 33) (9.09%). Moderate to high rates of resistance to azithromycin, erythromycin, cefoxitin and tetracycline were recorded across all isolates, ranging between 26.47–58.3%, 26.47–66.7%, 23.08–50%, 7.69–50%, respectively. Also, resistance to fusidic acid was common in the sample (52.91–93.94%). All isolates were susceptible to linezolid and resistance to rifampicin and vancomycin was detected only in a single *S. aureus* isolate each, whereas resistance to teicoplanin was detected in one *S. haemolyticus* isolate (Fig. 3, and Supplementary files 5 and 6).

Apart from *S. capitis* (*n* = 1), multi-drug resistance (MDR) was detected among all staphylococcal species, ranging from 30.77% among *S. hominis* to 50% among *S. epidermidis*. The only *S. lentus* isolate present in the study also displayed MDR (Fig. 3).

The susceptibility results were confirmed by MIC determination (Supplementary files 7 and 8). Azithromycin had the widest MIC range (1– > 1024 µg/ml for *S. aureus* and 0.5– > 1,024 µg/ml for SOSA isolates), while linezolid had the narrowest MIC range (0.5–1 µg/ml for *S. aureus* and SOSA), followed by vancomycin (0.25–2 µg/ml for *S. aureus* and 0.5–2 µg/ml for SOSA) (Table 2). Apart from fusidic acid where the highest MIC values recorded for SOSA were eightfold higher than *S. aureus* (1024 vs. 128 µg/ml), comparable highest and lowest MIC values in the range were detected for SOSA and S*. aureus*. MIC_50_ and MIC_90_ were determined using our dataset. At least four-fold elevated MIC_50_ values were observed for azithromycin against *S. haemolyticus* and *S. epidermidis* (32 µg/ml) and doxycycline against *S. epidermidis* (4 µg/ml). Elevated MIC_90_ values were observed for fusidic acid against *S. haemolyticus* (1024 µg/ml) and gentamicin against *S. haemolyticus*, *S. epidermidis* (MIC_90_ 8 µg/ml) and *S. aureus* (MIC_90_ 16 µg/ml) (Table 2). Of note, the MIC_90_ for doxycycline against *S. aureus* is four- to five-fold reduced (MIC_90_ 0.25 µg/ml) compared to the other species.

**Table 2:** MIC_50_ and MIC_90_ (µg/ml) of tested antibiotics against each staphylococcal species using agar dilution method.

	*S. aureus*	*S. haemolyticus*	*S. hominis*	*S. epidermidis*	*S. warneri*	*S. capitis*	*S. lentus*
Number of isolates	34	33	13	12	9	1	1
Azithromycin							
Total MIC Range	1– > 1024	0.5– > 1024	< 0.5– > 1024	< 0.5– > 1024	< 0.5– > 1024	1	128
MIC_50_	2	32	1	32	1	–	–
MIC_90_	> 1,024	> 1,024	> 1,024	1,024	512	–	–
Cefoxitin							
Total MIC Range	2–16	2–16	1–16	1–4	1–8	16	16
MIC_50_	4	4	4	2	4	–	–
MIC_90_	8	16	16	4	4	–	–
Chloramphenicol							
Total MIC Range	4–32	1–32	1–8	1–32	2–8	8	4
MIC_50_	4	4	2	4	4	–	–
MIC_90_	32	16	8	8	8	–	–
Doxycycline							
Total MIC Range	0.25–8	0.25–8	0.25–4	0.25–8	0.25–4	0.25	0.25
MIC_50_	0.25	1	0.25	4	0.25	–	–
MIC_90_	0.25	8	4	4	4	–	–
Fusidic acid							
Total MIC Range	< 0.5–128	< 0.5–1024	< 0.5–32	< 0.5–256	< 0.5–16	4	8
MIC_50_	2	8	2	16	8	–	–
MIC_90_	16	1,024	16	16	8	–	–
Gentamicin							
Total MIC Range	< 0.5–128	< 0.5–64	< 0.5–4	< 0.5–8	< 0.5–8	< 0.5	< 0.5
MIC_50_	< 0.5	< 0.5	< 0.5	< 0.5	< 0.5	–	–
MIC_90_	16	8	0.5	8	< 0.5	–	–
Linezolid							
Total MIC Range	0.5–1	0.5–1	0.5–1	0.5–1	0.5–1	0.5	0.5
MIC_50_	1	0.5	0.5	1	0.5	–	–
MIC_90_	1	1	1	1	1	–	–
Vancomycin							
Total MIC Range	0.25–2	1–2	0.5–2	0.5–2	0.5–2	1	1
MIC_50_	0.5	1	1	1	1	–	–
MIC_90_	1	2	2	2	2	–	–

### Individual colonization by MDR staphylococci

40/86 (46.51%) of the volunteers were found to be colonized by at least one MDR isolate, with 14/86 (16.28%) individuals carrying MDR *S. aureus* and 26/86 (30.2%) harboring MDR SOSA isolates. Four individuals carried more than one MDR isolate, two of them carried concomitantly MDR *S.* *aureus* and MDR SOSA, while the other two participants were colonized by different MDR-SOSA. No significant correlation existed between previous hospitalization and MDR carriage (*p* = 0.33; *ns*).

### Characterization of macrolide resistance

Regardless of phenotypic resistance exhibition, all isolates were tested for macrolide resistance gene carriage. Thus, presence of *ermA*, *ermB*, *ermC*, *ermT* and *msrA* genes was determined using PCR (Supplementary files 9 and 10). *ermB* showed the highest prevalence among all species (79.41% and 94.2% among *S. aureus* and SOSA, respectively), followed by *ermC* (38.24% and 86.96% among *S. aurues* and SOSA, respectively) (Table 3). Of note, prevalence of *ermB* and *ermC* was lower in *S. aureus* than in SOSA. *ermA* was detected in only one *S. epidermidis* isolate. On species level and excluding *S. lentus*, *S. epidermidis* had the highest rates of azithromycin resistance (58.33%) and the highest prevalence of resistance genes among all species, *ermB* (100%), *ermC* (91.67%), *msrA* (75%), and *ermA* and *ermT* (8.33%, each) (Table 3). Presence of *ermC*, *ermB* and *msrA* was significantly associated with *S. aureus* (*p* < 0.05). This holds also true for *ermC*, *msrA* and *S. haemolyticus* (*p* < 0.05). In *S. aureus*, azithromycin resistance was significantly associated with the presence of *msrA* (*p* < 0.05). *S. aureus* showed the highest percentage of D-test positive isolates (77.78%), followed by *S. haemolyticus* (57.89%). Constitutive MLS_B_ resistance was equally observed in *S. aureus* and SOSA (11.11% and 16.22%, respectively), whereas inducible MLS_B_ resistance was more often found in *S. aureus* (77.78% and 43.24%, respectively) (Table 4).

**Table 3:** Relationship between species and phenotypic and genotypic resistance to azithromycin.

Species *p* value	Phenotypic resistance n (%)	*ermC* n (%)	*ermB* n (%)	*ermA* n (%)	*ermT* n (%)	*msrA* n (%)
*S. aureus*	9 (26.5)	13 (38.2)	27 (79.4)	0 (0)	0 (0)	4 (11.8)
*p* value		** < 0.0001**^**a**^ 0.5^b^	**0.043**^**a**^ 0.35^b^	1^a^ 1^b^	0.55^a^ 1^b^	** < 0.0001**^**a**^ **0.048**^**b**^
*S. haemolycicus*	19 (57.6)	28 (84.8)	32 (97)	0 (0)	1 (3)	23 (70)
*p* value		**0.038**^**a**^ 1^b^	0.10^a^ 0.42^b^	1^a^ 1^b^	1^a^ 1^b^	**0.0015**^**a**^ 0.14^b^
*S. hominis*	6 (46.2)	11 (84.6)	11 (84.6)	0 (0)	0 (0)	7 (21.2)
*p* value		0.34^a^ 1^b^	0.34^a^ 0.46^b^	1^a^ 1^b^	1^a^ 1^b^	0.54^a^ 0.08^b^
*S. epidermidis*	7 (58.3)	11 (91.7)	12 (100)	1 (8.3)	1 (8.3)	9 (75)
*p* value		0.17^a^ 1^b^	0.35^a^ 1^b^	0.12^a^ 1^b^	0.31^a^ 1^b^	0.061^a^ 1^b^
*S. warneri*	4 (44.4)	8 (88.9)	8 (88.9)	0 (0)	1 (11.1)	4 (44.4)
*p* value		0.28^a^ 1^b^	0.54^a^ 1^b^	1^a^ 1^b^	0.24^a^ 0.44^b^	1^a^ 0.21^b^
*S. capitis*	0 (0)	1 (100)	1 (100)	0 (0)	0 (0)	0 (0)
*p* value		1^a^ 1^b^	1^a^ 1^b^	1^a^ 1^b^	1^a^ 1^b^	1^a^ 1^b^
*S. lentus*	1 (100)	1 (100)	1 (100)	0 (0)	0 (0)	1 (100)
*p* value		1^a^ 1^b^	1^a^ 1^b^	1^a^ 1^b^	1^a^ 1^b^	0.47^a^ 1^b^

^a^Refers to relationship between macrolide resistance gene and species (*p* values) at 0.05 significance level.

^b^Refers to relationship between presence of resistance genes and macrolide phenotypic resistance (*p* values) at 0.05 significance level.

Significant values are in bold.

**Table 4:** MLS_B_ inducibility among the isolates.

Species	Macrolide resistance (E-R, AZM-R)	MLS_B_ constitutive* (E-R, AZM-R + CD-R) (%)	MLS_B_ inducible (D-test positive) (E-R, AZM-R + CD-S) (%)	Macrolide-streptogramin B resistance (D-test negative) (E-R, AZM-R, CD-S) (%)	*erm* genes (*ermA*, *ermB*, *ermC*)	*msrA* gene
*S. aureus*	9/34	1/9 (11.11)	7/9 (77.78)	1/9 (11.11)	8/9	3/9
*S. haemolyticus*	19/33	1/19 (5.26)	11/19 (57.89)	7/19 (36.85)	19/19	15/19
*S. hominis*	6/13	0/6 (0)	2/6 (33.33)	4/6 (66.66)	6/6	5/6
*S. epidermidis*	7/12	3/7 (42.86)	3/7 (42.86)	1/7 (14.28)	7/7	5/7
*S. warneri*	4/9	2/4 (50)	0/4 (0)	2/4 (50)	4/4	2/4
*S. capitis*	0/1	–	–	–	1/1	0/1
*S. lentus*	1/1	0/1 (0)	0/1 (0)	1/1 (100)	1/1	1/1

*Additional lincosamide resistance (clindamycin). *E* erythromycin, *AZM* azithromycin, *CD* clindamycin, *R* resistant, *S* susceptible.

## Discussion

Health care-associated (HA) infections are a public health issue worldwide, including Egypt^25,26^ Data from Mansoura New General Hospital in 2017 showed that 3.7% of hospitalized patients experienced a HA infection during the study period^25^. In addition, community-acquired (CA) infections contribute to the disease burden as well. In this context, some bacteria circulating in the population, such as CA-MRSA, are particularly feared when they enter hospitals due to their high virulence potential^27,28^. A study from Egypt in 2020 reports that the leading cause of heart failure exacerbation in hospitalized patients with former heart failure were due to infections by CA-bacteria, with an in-hospital mortality rate of up to 7.7%^29^. One explanation why bacteria from the community are difficult to treat might be antibiotic resistance which in turn is often associated with the abuse and overuse of antibiotics outside of hospitals.

In this study, we investigated nasal carriage of staphylococci in young volunteers, in order to gain insight into the staphylococcal species circulating in individuals in an urban setting in Egypt, and their ABR profiles. From a total of 196 volunteers, we obtained 103 staphylococcal isolates from 86 people. The detected total *S. aureus* colonization rate of 34.88% (30/86) is not unusual and corresponds very well with previously reported numbers in similar cohorts^30^. However, an MRSA carriage rate of 14% (12/86) is remarkable and suggests that MRSA are widespread and circulate in the community in the Alexandria urban region^31^. Here, multi-locus sequence typing (MLST) upon whole genome sequencing (WGS) of the strains would be very useful in the future to identify the exact MRSA clonal lineages spreading in the region. With respect to SOSA, we found (as expected) a broad range of typical species with *S. haemolyticus*, *S. hominis* and *S. epidermidis* being the most common ones (Fig. 2). Methicillin resistance among SOSA was strikingly frequent, with 39.13% (27/69) of the isolates displaying cefoxitin resistance (as a proxy for methicillin resistance). In general, SOSA displayed higher antimicrobial resistance rates than *S. aureus* (Fig. 3A, C) which is in good agreement with previous studies^32,33^. The percentage of multidrug resistant (MDR) isolates among SOSA species was 43.48% (Fig. 3C) which is twice the rate reported in a recent German study in volunteers in the community^34^. The numbers detected in our study, rather resemble ABR rates reported previously for health care workers in various countries across the globe^35^. MDR rates for *S. aureus* were 41.18%, slightly lower than reported for non-hospitalized individuals in Nigeria (52.2%)^36^, but nearly three times the rate reported for health care workers in a global study^35^, suggesting again a high antibiotic selective pressure in the Alexandria region.

Regarding individual antibiotics, we found high rates of fusidic acid resistance in both *S. aureus* and SOSA (52.94% and 81.16%, respectively, which is in agreement with a previous study also reporting high levels of fusidic acid resistance in *S. aureus* (71.9%) and coagulase negative staphylococci (52.6%)^37^. The current prevalence of fusidic acid resistance is higher than the rate presented by our group (32.1%) in 2017 among a collection of staphylococci from various hospitals in Alexandria^38^. The high susceptibility levels detected here with vancomycin mirror the rates detected by our group among a collection of *S. aureus* and *S. haemolyticus* clinical isolates and selected mutants^39^. Macrolides are among the most commonly used antibiotics in outpatients in Egypt and worldwide^15,16^, and accordingly, we found high resistance rates for azithromycin (44.66%) and erythromycin (45.63%) in all staphylococcal species (Fig. 3C), with data matching well with previous studies^40^. We analyzed the prevalence of macrolide resistance genes present in the isolates by PCR and found them in most staphylococcal species with prevalence ranging between 8.3% and 100%, which agrees with the phenotypic data. *ermB* showed the highest prevalence (Table3), differently to what was reported before^41,42^, which could be explained on the basis of regional differences^43^. In Africa, *ermB* is more prevalent than in other regions, fitting with our observation. On the other hand, *ermA* was only detected in one *S. epidermidis* isotate, which agrees with the data from Africa too, where it was the least detected *erm* gene^43^. While phenotypic reistance data fit what was previouly reported, the prevalence of resistance genes in the current study is lower^41^. Additionally, D-test was perferomed to investigate prevalence of consitutive and inducible MLS_B_ resistance. Inducible phenotype was previously reported in staphylcococci at low ranges up to 30% which is less than we detected (*S. aureus*: 77.78% and SOSA: 43.24%)^23,40,44,45^. Constitutive MLS_B_ resistance was equally obersved in *S. aureus* and SOSA (11.11% and 16.22%, respectively). That is in concordance with published data, where consitivitve MLS_B_ resistance was reported up to 11%^23,44^. The high rates of inducible MLS_B_ resistance are very alarming, as this harbous a huge risk for treatment failure in case of an infection where treatment is attempted with clindamycin in the absence of D-test data.

Finally, all isolates showed complete susceptibility to linezolid which is in good agreement with previous studies^40,46^.

Analysis of the data set with regard to such criteria as previous diseases or hospitalization of the participants did not reveal any statistically robust correlation to colonization with specific species or (resistant) strains. Previous studies suggested that personal hygiene, more precisely nose ablution, may influence nasal microbiota^47,48^. However, we could not demonstrate such an association in our study (Table 1).

## Conclusions

Taken together our data show that in the Alexandria region (multidrug) resistant staphylococci are no longer restricted to hospital environments but are now also widespread in the community. It is tempting to speculate that the high MRSA and MDR detection rates observed are associated with high antibiotic selection pressure in the region. The alarming rates of inducible MLS_B_ resistance phenotypes among commensal isolates call for making D-test data available to infectious disease physicians alongside susceptibility data. Whether the detected high resistance rates are also coupled with high virulence rates is an important research question for future studies. A limitation of the study is that it only focused on staphylococcal carriage among pharmacy students and didn’t include any healthcare workers. In general, our study is among the very few studies on record of staphylococcal nasal colonization (both *S. aureus* and SOSA) among Egyptians. Future plans include conducting whole genome sequencing of the isolates for further elucidation of their resistance and virulence genotypes as well as a detailed description of the isolate epidemiology.

### Supplementary Information

Supplementary Information.

## Data Availability

The datasets generated and analysed in the current study are available from the corresponding author upon reasonable request.

## Abbreviations

*S. aureus*: *Staphylococcus aureus*
SOSA: Staphylococci other than *S. aureus*
*erm*: Erythromycin ribosomal methylase
*msrA*: Macrolide streptogramin resistance A
MDR: Multi-drug resistance
*S. hominis*: *Staphylococcus hominis*
*S. epidermidis*: *Staphylococcus epidermidis*
*S. haemolyticus*: *Staphylococcus haemolyticus*
*S. warneri*: *Staphylococcus warneri*
*S. lentus*: *Staphylococcus lentus*
*S. capitis*: *Staphylococcus capitis*
MLS_B_: Macrolide-lincosamide-streptogramin B
ABR: Antibiotic resistance
MRSA: Methicillin resistant *S. aureus*
DNase: Deoxyribonuclease
GPI: Gram-positive identification test
CLSI: Clinical and Laboratory Standards Institute
EUCAST: European Committee on Antimicrobial Susceptibility Testing
PCR: Polymerase chain reaction
SPSS: Statistical Package for Social Science
MSA: Mannitol salt agar
MIC: Minimum inhibitory concentration
HA: Health care-associated
CA: Community-acquired
MLST: Multi-locus sequence typing
WGS: Whole genome sequencing
CX: Cefoxitin
C: Chloramphenicol
CD: Clindamycin
VA: Vancomycin
TE: Tetracycline
MUP: Mupirocin
TEI: Teicoplanin
LZ: Linezolid
MO: Moxifloxacin
RIF: Rifampicin
COT: Co-trimoxazole
GEN: Gentamicin
FC: Fusidic acid
E: Erythromycin
AZM: Azithromycin
